# Risk factors for oral mucositis in paediatric oncology patients receiving alkylant chemotherapy

**DOI:** 10.1186/1472-6831-6-13

**Published:** 2006-10-18

**Authors:** Giulia Fadda, Guglielmo Campus, PierFranca Lugliè

**Affiliations:** 1 Dental Institute, University of Sassari, Viale San Pietro 43/c I-07100 Sassari, Italy; 2 Institut Gustave Roussy, Paris, France

## Abstract

**Background:**

We describe the risk indicators for oral mucositis (OM) in paediatric oncology patients hospitalised in the Institut Gustave Roussy (Villejuif-Paris) and treated with alkylant chemotherapy with autologous peripheral blood progenitor cells.

**Methods:**

The sample was selected using PIGAS software. Three groups of subjects received different chemotherapy regimens: A. Melphalan, B. Busulfan and C. other alkylant protocols. The degree of mucositis was recorded by CTC version 2.0 (Common Toxicity Criteria). Descriptive statistics were performed. The association between mucositis and risk indicator variables was tested using a χ^2 ^test. The association between case status and covariates was tested using unconditional logistic regression analysis.

**Results:**

Of the 337 children enrolled, 241 showed mucositis (group 1) and 96 did not show mucositis (group 2) during alkylant chemotherapy. There was a higher prevalence of male patients in both groups. The three different chemotherapy regimen groups are correlated with the appearance of oral mucositis (χ^2 ^= 22.42, p < 0.01). Weight loss was higher in group 1 (χ^2 ^= 6.31, p = 0.01). The duration of aplasia was lower in the Busulfan protocol (7.5 days) than in the Melphalan group (9.3 days) or the other regimens (8.6 days). The use of Bufulfan^® ^was directly associated with case status (presence of oral mucositis): odds ratio [OR] = 2.1 and confidence interval [95%CI] = 1.3–3.0. Also, occurrences of germinal tumours and secondary bacterial infections were directly linked with case status: [OR] = 1.4 and 1.8, confidence interval [95%CI] = 1.2 – 1.7 and 1.1 – 2.5, respectively.

**Conclusion:**

The presence of OM was associated with the three different chemotherapy regimens considered; in particularly patients treated with Busulfan had the highest prevalence.

## Background

Many forms of cancer can be treated effectively with radiotherapy and chemotherapy. However, these treatments have significant dose-limiting toxicities. In western countries, 1 out of every 500 to 600 children develops childhood cancer before reaching the age of 15 years [1]. Currently, high-dose chemotherapy with autologous peripheral blood progenitor cell transplantation is the primary treatment for tumoral lesions in children [2]. Cancer and chemotherapy are amongst the leading health problems influencing the quality of life of the individual. The complications of many treatment regimens appear frequently in the mouth and cause serious disturbances. Recent investigations have demonstrated that many serious infections originate from the mouth, and anti-neoplastic chemotherapy or immunosuppressive drugs increase the susceptibility of patients [3].

Oral mucositis (OM) is multifactorial in nature. The disruption or loss of rapidly dividing epithelial progenitor cells is a trigger for the onset of the disorder. However, the actual manifest dysfunction and its severity and duration are greatly influenced by changes in other cell populations, immune responses and the effects of oral flora. This toxicity frequently complicates the course of autologous bone marrow transplantation; it causes severe pain as well as cramping, nausea and gastro-enteritis. In addition, food and fluid intake may be poor, the ability to absorb nutrients much reduced and the susceptibility to infection greatly increased. The nature and degree of mucositis experienced by a patient varies according to the treatment regimen applied (combination of radiotherapy and chemotherapy, dosage, duration and sequence). Mucositis can therefore result in under-nutrition and significantly decreases a patient's quality of life. Modulation of the treatment regimen (use of lower doses or long recovery intervals between doses) remains the most effective means of limiting the actual incidence and severity. This event can therefore compromise patient prognosis [4].

Mucositis is an iatrogenic stomatitis that usually begins with aplasia, between 7 and 14 days after the initiation of chemotherapy. During the subsequent 1–2 weeks there is a loss of epithelial structure and integrity and severe ulceration develops. Much of this damage occurs in non-keratinized areas such as the cheeks, underside of the tongue and floor of the mouth. The epithelium of the oral cavity and of the digestive tract can renew rapidly, making their cells highly sensitive to the cytotoxic effects of chemotherapy. Mucosal lesions are only temporary. They are caused by the lowering of the renewal rate of the basal epithelium, leading to the thinning, denudation and ulceration of the soft tissue of the digestive system [5]. Epithelial denudation and mucosal damage may persist for 2–4 weeks after the cessation of radiotherapy [6]. The pathophysiology of this condition is still undefined, but recently the hypothesis has been put forward that mucositis is the result of a complex interaction of factors occurring in different phases [7].

All the regimens used in the Department of Paediatric Oncology of the Institut Gustave Roussy include alkylating agents. These agents are currently the most effective and frequently-used antineoplastic agents for treating paediatric cancers such as cerebral tumours (medulloblastoma, neuroblastoma) and other solid tumours such as osteosarcoma. They form covalent links with DNA. This may explain the anticarcinogenic power and cytotoxicity of these drugs. The effects on DNA are most marked in cells with a high mitotic index, including the proliferating tissues of the bone marrow and the lining of the gastrointestinal tract.

The aim of the present investigation was to identify risk indicators and side effects of oral mucositis in a paediatric population receiving alkylant chemotherapy. It is important to describe factors that affect mucositis in order to reduce the frequency of this side effect and to improve the quality of life of patients.

## Methods

### Study design

This study was designed as a retrospective cross-sectional case-control study. The study was approved by the Ethical Committee of the University of Sassari, Italy (n° 508/2004). The sample consisted of a paediatric oncology population from 1 to 15 years old, hospitalized in the Paediatrics Department ("La Mer") of the IGR from June 1992 to June 2003; during this period 453 subjects were examined. All patients were treated by HDC containing at least one alkylant drug in the protocol, followed by autologous stem cell transplantation and then by supplementation with granulocyte colony-stimulating factor. They all received conventional chemotherapy, either at diagnosis or at relapse or both, in accordance with on-going European Society for Medical Oncology protocols [8]. The conditioning regimens were categorised into three groups on the basis of the principal drug included in the protocol (Melphalan, Busulfan, other alkylant drug). During treatment, the patients underwent a standardized oral care regimen: mouthwashes twice a day throughout the treatment. Exclusion criteria included age greater than 15 years (72 patients), previous radiotherapy (36 subjects) or more than 2 autografts (8 subjects). If two bone marrow transplantations had occurred, only the first was considered.

### Methods

Patients' data were collected by the nursing and medical staff of IGR, transcribed on to standard forms, then entered on a database managed with PIGAS (Gustave Roussy Institute, Villejuif, France) [9]. The autologous stem cell transplantation conditioning regimens and supportive care regimens remained the same throughout the 10 years of data collection. OM was graded by the *Common Toxicity Criteria (CTC) version 2.0 *of the National Cancer Institute [10] as shown in Table 1. In this study, only grades greater than 1 were considered in relation to mucositis.

**Table 1:** Common Toxicity Criteria (CTC) version 2.0

**Grade 0**	No mucositis
**Grade 1**	Painless ulcers, erythema, or mild soreness in the absence of lesions
**Grade 2**	Painful erythema, oedema or ulcers, ability to eat solid
**Grade 3**	Painful erythema, oedema, or ulcers preventing swallowing or requiring hydration or parenteral (or enteral) nutritional support
**Grade 4**	Impossible to swallow

The sample was divided in two groups: children with (cases) and without (controls) oral mucositis. Gender and age were recorded at diagnosis and at the time of autologous stem cell transplantation. All children in the sample received HDC including at least one alkylant drug, and on this basis were divided into three groups: treated with Melphalan, with Busulfan, or with other alkylant agent.

Aplasia was defined as a white blood cell count < 500/mm^3^, red corpuscle count < 2,5 × 106/mm^3 ^and platelets < 20000/mm^3^. The duration of aplasia was recorded. Children were weighed on calibrated weighing scales and percentage weight loss was calculated. Viral and bacterial infections were diagnosed using cotton-tipped sterile swabs. Grades of vomiting, diarrhoea and anorexia were scored by the NCI/SIOP quotation system. Finally, nervous central system problems and haemorrhage were recorded (Table 2).

**Table 2:** Characteristics of cancer chemotherapy patients receiving alkylant agents

Gender		
	Group 1 (mucositis) n = 241 n (%)	Group 2 (no mucositis) n = 96 n (%)

Male	146 (60.5)	67 (69.7)
Female	95 (39.5)	29 (30.3)

χ^2 ^Mantel-Haenszel = 2.50 p = 0.11		

Diagnosis		

	Group 1 (mucositis) n = 241 n (%)	Group 2 (no mucositis) n = 96 n (%)

Cerebral tumors	70 (30.7)	27 (28.1)
Germinal tumors	78 (32.4)	22 (22.9)
Sarcoma	68 (28.2)	36 (37.5)
Hodgkin	9 (3.7)	11 (11.5)
Other	12 (5.0)	- (--)

χ^2 ^= 13.46 p < 0.01		

Chemotherapy Regimens		

	Group 1 (mucositis) n = 241 n (%)	Group 2 (no mucositis) n = 96 n (%)

Melphalan^®^	24 (9.9)	20 (20.8)
Busulfan^®^	153 (63.5)	34 (35.4)
Other	64 (26.6)	42 (43.8)

χ^2 ^= 22.42 p < 0.001		

### Statistical methods

STATA statistical data analysis software (Version 8.2, Stata Corporation, College Station, Texas USA) was used. Initially, clinical condition parameters and potential risk indicators were analysed univariately to describe the variables and distributions. Student's t test was used to compare the two groups, and p < 0.05 was taken as the criterion of significance. To avoid the attenuating effect of unequal variability among groups on the value of t, a square root transformation was performed when the response variable was a count. For categorical variables, difference between groups (1 and 2) was evaluated by χ^2 ^test or Fischer's exact test.

Possible associations of cases (patients with oral mucositis) or controls (patients without oral mucositis) with treatment regimen, aplasia and other variables were analysed using unconditional logistic regression analysis.

Covariates resulted statistically significant in bivariate analysis entered in the logistic regression.

## Results

There were 337 patients in the analytical sample (213 boys and 124 girls): 241 in the case group (with mucositis, grade > 1) and 96 in the control group (without mucositis, grade ≤ 1). The mean age was 7.6 years at the time of diagnosis and 8.9 years at the time of autologous stem cell transplantation. Table 2 describes the characteristics of the patients included in this study and their treatments. There was a higher prevalence of male patients in both groups (χ^2 ^= 2.50, *p *= 0.11). Tumours were divided into five groups: cerebral tumours (CT) (n = 97, 28.8%), germinal tumours (n = 100, 29.7% including neuroblastoma, nephroblastoma and retinoblastoma), sarcoma (n = 104, 30.9% including osteosarcoma, Ewing's sarcoma and rhabdomyosarcoma), Hodgkin tumours (n = 20, 5.9% including Hodgkin disease and non-Hodgkin lymphoma) and miscellaneous (n = 12, 3.6%). Most of the tumoral pathologies were correlated with expression of oral mucositis (χ^2 ^= 13.46, *p *< 0.01), especially embryonic carcinoma (100%), nephroblastoma (100%) and neuroblastoma (75.3%). The mean ages at the times of diagnosis (7.6 ± 5.4 years for group 1 and 7.5 ± 5.6 years for group 2, *t *= -0.06, *p *= 0.84) and autologous stem cell transplantation (9.2 ± 6.9 years for group 1 and 8.8 ± 6.3 years for group 2, *t *= -0.21, *p *= 0.95) were similar in both groups (data not in tables).

**Figure 1:**
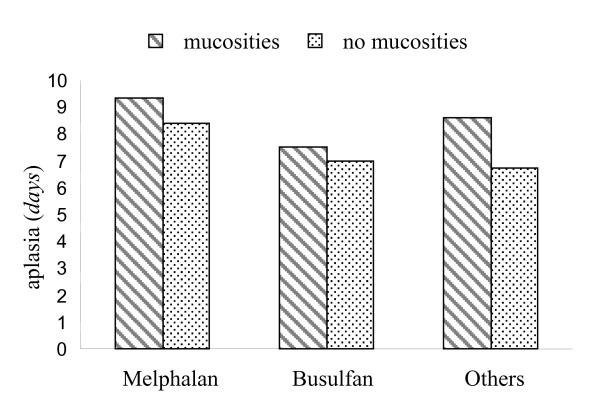
Mean duration of aplasia following different chemotherapy regimens.

The three different chemotherapy regimens were associated with the appearance of oral mucositis (χ^2 ^= 22.42, *p *< 0.01). The patients treated with Busulfan had the highest prevalence of mucositis (153 subjects, 63.5%). The association of various predictor variables with the consequences of oral mucositis is shown in Table 3. Weight loss was higher in group 1 and lower in the other group (χ^2 ^= 6.31, *p *= 0.01). Anorexia was present more frequently in patients who developed mucositis, *i.e*. anorexia grade three was observed in 115 subjects with mucositis *versus *12 without mucositis (χ^2 ^= 68.01, *p *< 0.001). Most patients (83.2% in group 1 and 89.5% in group 2) did not develop viral infections during the treatment (χ^2 ^= 2.09, *p *= 0.10), while bacterial infections were noticed in 97.8% of the subjects in group 1 and 81.3% in group 2 (χ^2 ^= 29.93, *p *< 0.01). The occurrence of vomiting was higher in group 1 and considerably lower in group 2 (χ^2 ^= 31.73, *p *< 0.001). Cerebral nervous system complications and haemorrhage were not associated with the occurrence of OM (χ^2 ^= 1.69, *p *= 0.19 and χ^2 ^= 2.09, *p *= 0.15, respectively). The mean duration of aplasia was 8.4 days in group 1 and 7.4 days in group 2 (*t *= -2.27 *p *= 0.02). Three members of the first group did not report these data, therefore the analysis was performed on 334 patients. The duration of aplasia estimated in relation to the three different chemotherapy regimens (334 children) is displayed in figure 1. It was lower in the Busulfan protocol (7.5 days); in the other chemotherapies the durations were 9.3 days (Melphalan) and 8.6 days (other conditioning regimens).

**Table 3:** Experience of oral mucositis by risk indicators as count (percentage) weight loss

	Group 1 (mucositis) n = 241 n (%)	Group 2 (no mucositis) n = 96 n (%)
Yes	142 (58.9)	43 (44.8)
No	95 (39.5)	53 (55.2)

χ^2 ^Mantel-Haenszel = 6.31 p = 0.01		

Anorexia		

	Group 1 (mucositis) n = 241 n (%)	Group 2 (no mucositis) n = 96 n (%)

Grade 0	5 (2.1)	14 (14.6)
Grade 1	22 (9.1)	31 (32.3)
Grade 2	99 (41.1)	32 (33.3)
Grade 3	115 (47.7)	12 (12.5)

χ^2 ^= 68.01 p < 0.001		

Viral infections		

	Group 1 (mucositis) n = 241 n (%)	Group 2 (no mucositis) n = 96 n (%)

Yes	40 (16.8)	10 (10.5)
No	198 (83.2)	85 (89.5)

χ^2 ^Mantel-Haenszel = 2.09 p = 0.15		

Bacterial infections		

	Group 1 (mucositis) n = 241 n (%)	Group 2 (no mucositis) n = 96 n (%)

Yes	236 (97.8)	78 (81.3)
No	5 (2.1)	18 (18.8)

χ^2 ^Mantel-Haenszel = 29.93 p < 0.001		

Vomiting		

	Group 1 (mucositis) n = 241 n (%)	Group 2 (no mucositis) n = 96 n (%)

Grade 0	36 (14.9)	33 (34.4)
Grade 1	60 (24.9)	31 (32.3)
Grade 2	99 (41.1)	16 (16.7)
Grade 3	46 (19.1)	8 (8.3)

χ^2 ^= 31.73 p < 0.001		

Central nervous system complications		

	Group 1 (mucositis) n = 241 n (%)	Group 2 (no mucositis) n = 96 n (%)

Yes	23 (9.5)	5 (5.2)
No	218 (90.5)	91 (94.8)

χ^2 ^Mantel-Haenszel = 1.69 p = 0.19		

Haemmorhage		

	Group 1 (mucositis) n = 241 n (%)	Group 2 (no mucositis) n = 96 n (%)

Yes	34 (14.1)	8 (8.3)
No	207 (85.9)	88 (91.7)

χ^2 ^Mantel-Haenszel = 2.09 p = 0.15		

Table 4 presents the crude odds ratio (OR) estimates and the associated 95% confidence intervals for the association between case status (presence of oral mucositis) and the covariates examined. The use of Bufulfan^® ^was directly associated with the case status (presence of oral mucositis): [OR] = 2.1 and confidence interval [95%CI] = 1.3–3.0. Also, occurrences of germinal tumours and secondary bacterial infections were directly linked with the case status: [OR] = 1.4 and 1.8, confidence interval [95%CI] = 1.2 – 1.7 and 1.1 – 2.5, respectively.

**Table 4:** Undiconditional logistic regression

	OR	95%CI
*Busulfan^®^*	2.1	1.3–3.0
*Germinal tumors*	1.4	1.2–1.7
*Bacterial infections*	1.8	1.1–2.5

## Discussion

In this retrospective analysis of 337 children treated with alkylant chemotherapy and autologous BMT, a considerable percentage of the patients experienced OM during treatment (group 1). The prevalence of oral mucositis is estimated to range from 30% to 99% [4,11-14]. However, most research into OM has been conducted on adult populations with few data on young populations, and for that reason we focused our attention on a paediatric group. Risk factors have not been clearly identified. Potential risk factors include genetic polymorphisms, gender, body mass, pre-existing oral condition, quantitative and qualitative salivary alterations and mechanical trauma induced by mastication [4,15]. Our study confirms that mucositis may be an underestimated complication in an oncological paediatric population. Gender and age were not identified as risk factor for OM; nevertheless, some previous studies have indicated that the female gender constitutes a significant factor risk for OM [16-18].

A systematic review of the research literature identified a vast number of interventions that have been evaluated for the prevention or treatment of oral mucositis in cancer patients. However, it is obvious that many interventions used in clinical practice have never been rigorously evaluated. Furthermore, many combinations of agents are advocated by local experts without evidence to support their use. Combining results from different studies during the systematic review and meta-analysis was limited, mostly because of differences among the study participants, interventions, and the timing and method of measuring outcomes. While many interventions used for treating or preventing mucositis have some evidence to support their use, no intervention has been conclusively validated by research [19,20].

Our results showed that almost every tumoral pathology is associated with evidence of OM except for rhabdomyosarcoma.

Most articles suggest a difference in severity grading between OM during chemotherapy and OM in the course of radiotherapy [21-23]. In our sample, the three groups of alkylating agents (with Melphalan, with Busulfan and with other drugs) were differently related to the manifestation of OM: the Busulfan regimen was associated with a greater risk of oral mucositis than the other alkylant agents. Several articles discuss the incidence and gravity of OM during alkylant regimens. Wardley and co-workers [4] reported that Melphalan protocols (High Dose Melphalan and High Dose Melphalan-Total Body Irradiation) and regimens involving Busulfan were associated with a grave mucositis (grade 3, WHO). No subject enrolled in our survey underwent total body irradiation. Rapoport and co-workers [24] indicated that Busulfan protocols may cause more severe OM then the others regimens.

The duration of aplasia was statistically lower in the affected group. Some studies [24,25] have reported an association between white cells and OM and confirmed the presence of white cells in the pathogenesis of mucositis. Other investigators [4] failed to find a statistically significant relationship between stomatitis and aplasia. Interestingly, we observed a shorter duration of aplasia in Busulfan regimens than in Melphalan protocols (or in the other chemotherapies). Our result may seem contradictory because the Busulfan protocol led to a higher percentage of stomatitis. Probable explanations of this inconsistency are:

1) the increased duration of aplasia in the Melphalan regimens (9.3 days) was caused by the use of VP-Carbo-Melph, a very strong chemotherapy protocol;

2) prolongation of the period of aplasia in alkylant regimens other than Melphalan or Busufan was attributable to the high frequency of patients with an osteosarcoma diagnosis treated with Tiothepa at a dose of 900 mg/m^2^; osteosarcoma patients do not accept bone marrow transplants very well, and this has a negative effect on the reduction of the duration of aplasia.

The diminution of the period of aplasia in the Busulfan protocol results from the proportionate augmentation of the duration in the other chemotherapy treatments.

We also investigated the relationship between the appearance of OM and other side effects of chemotherapy. Stomatitis has a statistically significant relationship with weight loss, anorexia, vomiting and bacterial infections. Each of the above-mentioned complications is a direct consequence of OM (weight loss and anorexia), or is related to a direct or indirect mucosal toxicity of the chemotherapy (vomiting and bacterial infections).

## Conclusion

We observed that the presence of OM was associated with the three different chemotherapy regimens considered; in particularly patients treated with Busulfan had the highest prevalence. We therefore propose as far as possible to decrease the use of Busulfan for treating paediatric cancers and to encourage the use of other chemotherapy regimens to improve patients' quality of life.

## Competing interests

The author(s) declare that they have no competing interests.

## Authors' contributions

GF contributed substantially to the conception and design of the study and carried out the acquisition of data;

GC participated in the design of the study, performed the statistical analysis and was involved in drafting the manuscript;

PFL conceived of the study and participated in its design.

All authors read and approved the final manuscript.

## Pre-publication history

The pre-publication history for this paper can be accessed here:


